# Multi-level personalization of neuromusculoskeletal models to estimate physiologically plausible knee joint contact forces in children

**DOI:** 10.1007/s10237-022-01626-w

**Published:** 2022-10-13

**Authors:** Giorgio Davico, David G. Lloyd, Christopher P. Carty, Bryce A. Killen, Daniel Devaprakash, Claudio Pizzolato

**Affiliations:** 1 Department of Industrial Engineering, Alma Mater Studiorum – University of Bologna, Bologna, Italy; 2 Medical Technology Lab, IRCCS Istituto Ortopedico Rizzoli, Bologna, Italy; 3 School of Allied Health Sciences and Social Work, Griffith University, Gold Coast, Australia; 4 Griffith Centre of Biomedical and Rehabilitation Engineering (GCORE), Menzies Health Institute Queensland, Griffith University, Gold Coast, Australia; 5 Department of Orthopaedics, Queensland Children’s Hospital, Children’s Health Queensland Hospital and Health Service, Brisbane, Australia; 6 Department of Movement Sciences, KU Leuven, Leuven, Belgium

**Keywords:** EMG, Joint contact forces, Musculoskeletal modeling, Pediatrics, Personalization

## Abstract

Neuromusculoskeletal models are a powerful tool to investigate the internal biomechanics of an individual. However, commonly used neuromusculoskeletal models are generated via linear scaling of generic templates derived from elderly adult anatomies and poorly represent a child, let alone children with a neuromuscular disorder whose musculoskeletal structures and muscle activation patterns are profoundly altered. Model personalization can capture abnormalities and appropriately describe the underlying (altered) biomechanics of an individual. In this work, we explored the effect of six different levels of neuromusculoskeletal model personalization on estimates of muscle forces and knee joint contact forces to tease out the importance of model personalization for normal and abnormal musculoskeletal structures and muscle activation patterns. For six children, with and without cerebral palsy, generic scaled models were developed and progressively personalized by (1) tuning and calibrating musculotendon units’ parameters, (2) implementing an electromyogram-assisted approach to synthesize muscle activations, and (3) replacing generic anatomies with image-based bony geometries, and physiologically and physically plausible muscle kinematics. Biomechanical simulations of gait were performed in the OpenSim and CEINMS software on ten overground walking trials per participant. A mixed-ANOVA test, with Bonferroni corrections, was conducted to compare all models’ estimates. The model with the highest level of personalization produced the most physiologically plausible estimates. Model personalization is crucial to produce physiologically plausible estimates of internal biomechanical quantities. In particular, personalization of musculoskeletal anatomy and muscle activation patterns had the largest effect overall. Increased research efforts are needed to ease the creation of personalized neuromusculoskeletal models.

**Supplementary Information:**

The online version contains supplementary material available at 10.1007/s10237-022-01626-w.

## Introduction

Neuromusculoskeletal (NMSK) models are digital and mathematical representations of the human anatomy and physiology, wherein bones are commonly described as rigid bodies interconnected by joints and actuated by muscles. When combined with motion capture and ground reaction force data, NMSK models can be used for biomechanical analyses of movement in healthy and pathological populations. By predicting and estimating internal biomechanical quantities that are difficult or invasive to measure in vivo (e.g., muscle and joint contact forces—JCFs), NMSK models may enable a better understanding of the mechanisms underpinning neuromuscular disorders (e.g., cerebral palsy—CP) (Steele et al. 2012a; Davico et al. 2020b), thus assisting clinical decision making. As such, NMSK models must provide a valid and accurate description of the neural and musculoskeletal systems. However, the generation of personalized models is not trivial and linearly scaled generic models are often used to approximate musculoskeletal anatomy.

Linearly scaled generic models rely on several assumptions and simplifications that might affect their estimates, possibly casting doubts on their ability to inform clinical decision making (Killen et al. 2020b). Musculoskeletal (MSK) anatomy is commonly linearly scaled from template models (Delp et al. 1990); however, musculotendon (MTU) parameters, such as tendon slack and optimal fiber length, do not linearly scale with bones or muscle lengths (Ward et al. 2005, 2007). Hence, the modelled muscles may show a not-physiological behavior (e.g., low or excessive activation levels, abnormal muscle force profiles), which strongly depends on the above parameters (Buchanan and Shreeve 1996; Winby et al. 2008; Carbone 2016). Generic bony geometries poorly represent real bones (Suwarganda et al. 2019; Bahl et al. 2019), possibly resulting in the inappropriate location of joint centers, particularly at the hip (Kainz et al. 2017b; Bahl et al. 2019), thus affecting joint angle calculations (Kainz et al. 2017a) and subsequent joint moments estimates (Bosmans et al. 2014). The location of muscle attachments may also be inaccurate due to morphological differences between generic and real bones, consequently altering MTU kinematics (Lenaerts et al. 2008; Scheys et al. 2008) and muscle forces estimates. Lastly, muscle activations are commonly synthesized using optimization algorithms, e.g., static optimization, under the assumption that the central nervous system favors a neural solution that minimizes the metabolic cost (Crowninshield and Brand 1981). However, this assumption may not be valid in individuals with neuromuscular pathologies who present with co-contractions and abnormal muscle activity (Brunner and Romkes 2008).

Several NMSK models with different levels of complexity and personalization (Table 1) have been developed over the years (Arnold* and Delp 2005; Steele et al. 2012a; Gerus et al. 2013; Brito da Luz et al. 2017; Fox et al. 2018; Modenese et al. 2018; Hoang et al. 2018; Wesseling et al. 2019; Veerkamp et al. 2019; Saxby et al. 2020). Accurate skeletal anatomies have been reconstructed from computed tomography (CT) or magnetic resonance imaging (MRI) scans via manual or semiautomatic segmentations (Brito da Luz et al. 2017) or generated via statistical shape modelling (Zhang et al. 2014; Nolte et al. 2016; Suwarganda et al. 2019; Davico et al. 2020a) and morphing techniques (Oberhofer et al. 2019). Muscle attachments, which may not be visible on medical images, have been morphed to personalized geometries using atlas-based approaches (Pellikaan et al. 2014), while via-points or wrapping surfaces (Modenese et al. 2018; Wesseling et al. 2019) were manually inserted to constrain muscle pathways. Optimal fiber and tendon slack lengths, which cannot be readily measured in vivo (Herzog et al. 1991), have been (1) tuned to ensure physiological muscle behavior (Winby et al. 2008; Modenese et al. 2016) and/or (2) calibrated within physiological ranges (Pizzolato et al. 2015; Serrancolí et al. 2016; Hoang et al. 2018). The maximal isometric force of muscles has often been scaled as a function of participants’ mass and/or height (Correa and Pandy 2011; Steele et al. 2012b; van der Krogt et al. 2016; Noble et al. 2017) or with segmented muscle volumes when medical images were available (Modenese et al. 2018; Davico et al. 2020b). Nonetheless, muscle weakness and altered mechanical properties due to neuromuscular disorders have commonly been based on literature data (Fox et al. 2009, 2018; Steele et al. 2012a). Finally, several methods were proposed to synthesize muscle activation patterns. These included dynamic optimization (Anderson and Pandy 2001), computed muscle control (Thelen et al. 2003), electromyography (EMG)-informed approaches (Lloyd and Besier 2003; Sartori et al. 2012; Pizzolato et al. 2015), synergy-based methods (Meyer et al. 2017) and stochastic approaches (van Veen et al. 2020). To the authors’ knowledge, only one work (Pitto et al. 2019) proposed ways to comprehensively incorporate different personalized features in a single NMSK model, but did not investigate the effects of personalization on muscle forces and JCF estimates. Therefore, the combined effect of different levels of personalization on muscle and knee JCF estimates remains unclear.

**Table 1:** Personalization methods. Methods employed to personalize musculoskeletal (MSK) anatomy, optimal fiber length (OFL), tendon slack length (TSL), maximal muscle isometric force (*F*_iso_^max^) and muscle activation patterns in neuromusculoskeletal models, using experimental, literature or models data

MSK anatomy	OFL&TSL	*F*_iso_^max^	Muscle activations
Segmentation of medical images (Brito da Luz et al. 2017) (bones&muscles)	Morphometric scaling (Modenese et al. 2016)	Scaling with mass (van der Krogt et al. 2016; Modenese et al. 2018)	Dynamic optimization (Anderson and Pandy 2001)
Statistical shape modelling (Zhang et al. 2014) (bones)	Calibration (Hoang et al. 2018)	Scaling with height (Steele et al. 2012b)	Computed muscle control (Thelen et al. 2003)
Mesh fitting techniques (Oberhofer et al. 2019) (bones)	Experimental data* (Gerus et al. 2015)	Scaling with muscle volumes (Modenese et al. 2018; Davico et al 2020b)	EMG-informed approaches (Pizzolato et al. 2015)
Atlas-based approaches (Pellikaan et al. 2014) (muscles)	–	Scaling with mass and length (Correa and Pandy 2011)	Synergy-based methods (Meyer et al. 2017)
Via-points (Modenese et al. 2018) (muscles)	–	–	Stochastic approach (van Veen et al. 2020)
Wrapping surfaces (Killen et al. 2020a) (muscles)	–	–	–

*EMG* Electromyography. Note that this table provides examples of methods employed to personalize MSK models, but it is not exhaustive

*Experimental data refer to the use of medical imaging data (e.g. ultrasounds) to determine muscle–tendon unit parameters

In this study, we developed six different NMSK models with incremental levels of personalization. The first aim was to determine which model was physiologically more plausible (i.e., better represented the underlying anatomical and physiological characteristics of each individual’s musculoskeletal system). The second aim was to quantify the individual effect of personalized (i) MTU parameters, (ii) neural solution, and (iii) musculoskeletal anatomy on the outcome variables. We hypothesized that a higher level of model personalization would correspond to more physiologically plausible estimates. Additionally, we hypothesized that personalizing the neural solution and musculoskeletal anatomy would affect model estimates more than calibrating MTU parameters.

## Material and methods

### Participants

Three children with unilateral CP (Gross Motor Function Classification System I-II. Age: 8.35 ± 2.01 years, height: 1.25 ± 0.12 m, mass: 23.30 ± 5.33 kg) and three typically developing (TD) age- and size-matched control participants (age: 7.98 ± 1.75 years, height: 1.23 ± 0.10 m, mass: 24.47 ± 6.02 kg) were enrolled in the study (Table 2). The study was approved by the Children’s Health Queensland Hospital and Health Services Human Research Ethics Committee, and informed consent was provided by each participant’s guardian or parent.

**Table 2:** Demographics of patients

Subject	Age (years)	Height (m)	Mass (kg)	Diagnosis	GMFCS
TD01	10.45	1.385	32.90	–	–
TD02	6.55	1.170	21.30	–	–
TD03	6.96	1.160	19.00	–	–
CP01	6.50	1.135	18.00	Hemiplegic	I
CP02	11.16	1.425	30.60	Diplegic	II
CP03	7.39	1.195	21.30	Diplegic	I

Demographics of the studied population, which included three children with cerebral palsy (CP) and 3 age-matched typically developing (TD) controls. *GMFCS* Gross Motor Function Classification System

### Gait analysis data collection and processing

A 3D gait analysis assessment was performed in the gait laboratory at Queensland Children Motion Analysis Service (QCMAS. Brisbane, QLD, Australia), where an experienced physiotherapist placed 51 retro-reflective motion capture markers on anatomical landmarks and twelve wireless bi-polar EMG sensors (Zerowire, Aurion, Milan, IT. 1000 Hz) on selected muscles of the right or (most) impaired lower limb (Davico et al. 2020b). Upon preparation, the correct placement of all EMG sensors was assessed by asking the participants to perform a series of exercises, each eliciting the activation of specific muscles (groups).

The gait laboratory was equipped with a 10-camera motion capture system (Vicon Motion System, Oxford, UK. 100 Hz) to record 3D marker trajectories, and 4 in-ground force platforms (Advanced Mechanical Technology Inc., Watertown, MA, USA. 1000 Hz) to simultaneously measure the ground reaction forces, while the participants performed dynamic tasks (i.e., overground walking trials). An additional static trial was collected, while the participants were standing in a T-pose.

For each participant, experimental data from 14 walking trials where clean foot strikes and good quality EMG data (i.e., minimally affected by noise) had been recorded were selected and further analyzed. Motion capture data were cleaned and labelled in Vicon Nexus 2.6 and then processed in MATLAB using the MOtoNMS toolbox (Mantoan et al. 2015). Both marker trajectory and ground reaction force data were filtered using fourth-order 6-Hz low-pass Butterworth zero-lag filter. EMG signals were band-pass-filtered (zero-lag double-pass second-order Butterworth, 30–400 Hz), full-wave rectified, low-pass-filtered (zero-lag double-pass second-order Butterworth, 6 Hz) and then normalized to each muscle’s maximal excitation identified across walking, heel raising and jumping tasks (Devaprakash et al. 2016).

### MRI imaging and processing

Following gait analysis, participants had MRI scans taken at the Queensland Children’s Hospital (QCH. Brisbane, QLD, Australia). All the motion capture markers placed on anatomical landmarks were replaced by MRI compatible markers, filled with a liquid visible on MRI, while the remaining upper body and cluster markers were removed. Full lower limb MRI scans, i.e., from the iliac crests to the malleoli, were acquired on a 1.5-T SIEMENS MAGNETOM Avanto fit syngo MR VE11B scanner (SIEMENS, Germany) using a 3D PD SPACE sequence (slice thickness: 1.0 mm, spatial resolution: 0.83 × 0.83mm^2^). Lower limb bony geometries, including pelvis, femurs, patellae, tibiae and fibulae, were manually segmented on the MRI scans using the Mimics Research Innovation Suite (v19).

### Base musculoskeletal model

The gait2392 OpenSim model (Delp et al. 1990) was selected as base musculoskeletal anatomy. The base model included 34 MTUs per leg (Sartori et al. 2012), a single degree-of-freedom (DOF) knee mechanism with generic splines defining knee flexion/extension and a two-point contact knee mechanism for the calculation of knee JCFs (Saxby et al. 2016). Medial and lateral contact points were positioned on the tibial plateaus based on a regression equation (Winby et al. 2009).

To match each participant’s size, the base musculoskeletal anatomy was linearly scaled with motion capture data according to established recommendations (Kainz et al. 2017b). However, since the population under study was significantly shorter and smaller than the subjects represented by the generic OpenSim gait2392 model, linearly scaled MTU parameters were further tuned. Specifically, we employed a morphometric scaling (Modenese et al. 2016) to optimize optimal fiber and tendon slack length values, thus ensuring a physiological muscle behavior throughout the full range of motion. Maximal isometric force values were scaled based on each participant’s mass, using the formula proposed by van der Krogt and colleagues (van der Krogt et al. 2016), which was specifically developed to better estimate maximal muscle force values in typically developing pediatric populations and children with CP (who tend to present with reduced muscle volumes) (Veerkamp et al. 2019):

1 $$F_{{{\text{SS}}}}^{{{\text{max}}}} = F_{G}^{{{\text{max}}}} \left( {{\raise0.7ex\hbox{${m_{{{\text{SS}}}} }$} \!\mathord{\left/ {\vphantom {{m_{{{\text{SS}}}} } {m_{G} }}}\right.\kern-\nulldelimiterspace} \!\lower0.7ex\hbox{${m_{G} }$}}} \right)^{{{\raise0.7ex\hbox{$2$} \!\mathord{\left/ {\vphantom {2 3}}\right.\kern-\nulldelimiterspace} \!\lower0.7ex\hbox{$3$}}}}$$

where ${m}_{\mathrm{SS}}$ is the mass of the participant, while ${m}_{G}$ and ${F}_{G}^{\mathrm{max}}$ are the mass and maximal isometric force values from the unscaled template model, respectively.

### Model personalization

The base model was progressively personalized to feature image-based anatomies, tuned and calibrated MTU parameters and muscle activation patterns derived from EMG data.

Personalized bony geometries were reconstructed combining statistical shape modelling and morphing techniques, providing full bone segmentations as input to maximize reconstruction accuracy (i.e., pipeline 9 in (Davico et al. 2020a)), and were generated using the free and open-source software Musculoskeletal Atlas Project (MAP) Client (Zhang et al. 2014). Personalized bones were then employed to customize a generic gait2392 OpenSim model (Delp et al. 1990) for use in biomechanical simulations. Feet bone geometries, for which a statistical shape model was not available in the MAP Client, were linearly scaled from the generic gait2392 model. During the customization process, the pathways of all muscles spanning the knee joint were also personalized. Muscle origin and insertion points, whose location on the mean (statistical shape) bone models was based on the SOMSO anatomical model (Marcus Sommer SOMSO Modelle, Sonneberg, Germany), were morphed together with the bone meshes. A set of wrapping surfaces (i.e., analytical shapes) were then fitted to selected anatomical regions to constrain muscle pathways (Table S1 in Online Resources), thus avoiding in-bone muscle penetrations and ensuring smooth MTU kinematics throughout the range of motion, via an automated process (Killen et al. 2020a). A visual check was finally performed to establish whether MTU kinematics presented any discontinuities; hence, whether manual adjustments (in either size or orientation of the wrapping surfaces) were required. The resulting MAP client-generated models featured personalized bones and physiologically and physically plausible muscle pathways. The knee joint was modelled as a 1-DOF hinge joint mechanism, where anterior/posterior and superior/inferior translations were prescribed to preserve a fixed distance between femur and tibia; the patellar body was locked to and moved along with the tibial body, via a fixed patellofemoral joint. Hip and ankle joints were defined as in the generic gait2392 OpenSim template model. A two-point contact knee mechanism was finally implemented to enable the estimation of knee JCFs (Winby et al. 2009; Saxby et al. 2016). In this case, medial and lateral contact points were positioned on the tibial plateaus where femur and tibia, with the model in default position (i.e., all joint angles equal to 0 degrees, with initial bone alignment based on the MRI data), were the closest.

Scaled and tuned MTU parameters were further adjusted via a calibration process in the Calibrated EMG-informed Neuromusculoskeletal Modelling Toolbox (CEINMS) (Sartori et al. 2012; Pizzolato et al. 2015), which employed experimental EMG signals to constrain values within physiological limits. The aim was to improve each model’s ability to track experimental joint moments and EMG data. During calibration, which was performed using four (out of fourteen) of the processed trials and across four degrees of freedom (i.e., ankle plantar/dorsiflexion, knee and hip flexion/extension, and hip ab/adduction), optimal fiber and tendon slack length values could vary by ± 5% from their original value. The strength coefficients that were used in CEINMS to control the force producing capability of each muscle group were bound between 0.5 and 1.5. For two subjects (TD03 and CP01) who were twins, as well as the shortest and lightest participants in the studied population, a ± 10% variation was implemented to ensure physiological muscle function. The more different the subjects were from the template models (in size, weight, and any other anthropometric characteristics), the more likely for the MTU parameters (even if scaled, tuned and calibrated) to be less close to the real values. Thus, resulting in abnormal muscle behavior, i.e., aberrant and excessive muscle excitations and force profiles, compared to experimental EMG and literature data—even for the TD participant.

Two different neural control solutions were employed in CEINMS to resolve the muscle redundancy problem: static optimization and EMG-assisted approach (Pizzolato et al. 2015; Hoang et al. 2018). A summary of the six developed NMSK models is presented in Table 3.

**Table 3:** Summary of the developed models

Model name	MSK anatomy	OFL & TSL	F_max_^iso^	Muscle activations
unCal_SO_	✘	Morph. Scaling (Modenese et al. 2016)	Scaled with mass (van der Krogt et al. 2016)	✘
Cal_SO_	✘	Morph. Scaling + Calibration (Pizzolato et al. 2015; Modenese et al. 2016; Hoang et al. 2018)	Scaled with mass	✘
Cal_EMGa_	✘	Morph. Scaling + Calibration	Scaled with mass	EMG-assisted approach (Pizzolato et al. 2015)
unCal_SO_^MAP^	MRI-based, SSM (bones) (Zhang et al. 2014)	Morph. Scaling	Scaled with mass	✘
Cal_SO_^MAP^		Morph. Scaling + Calibration	Scaled with mass	✘
Cal_EMGa_^MAP^	Atlas-based morphing, wrapping surfaces (muscles) (Killen et al. 2020a)	Morph. Scaling + Calibration	Scaled with mass	EMG-assisted approach

List of the neuromusculoskeletal models developed in this study, and their features.* EMG*  electromyography,* Morph. Scaling * morphometric scaling,* MRI* magnetic resonance images,* MSK* musculoskeletal,* OFL*  optimal fiber length,* SSM* statistical shape modelling,* TSL* tendon slack length. Symbols denote generic anatomies and MTU parameters linearly scaled from the gait2392 model or the use of static optimization to solve for muscle activations

### Biomechanical simulations

Biomechanical simulations were performed on both generic and personalized musculoskeletal anatomies in OpenSim (v 3.3) (Delp et al. 2007), where joint angles, joint moments and MTU kinematics were calculated using the inverse kinematics, inverse dynamics, and muscle analysis tools, respectively. OpenSim’s results were then provided as input for the CEINMS toolbox to guide the calibration process, when required, and to estimate muscle forces using the two neural control solutions, i.e., static optimization and EMG-assisted approaches. In both cases, the following objective function was solved frame by frame:

2 $$f = \alpha E_{{{\text{Moment}}}} + \beta E_{{{\text{sumEXC}}}} + \gamma E_{{{\text{EMG}}}}$$

where ${E}_{\mathrm{sumEXC}}$ is the sum of squared muscle excitations; ${E}_{\mathrm{Moment}}$, and ${E}_{\mathrm{EMG}}$ are the errors between experimental and estimated joint moments and muscle excitations, respectively; α, β and γ are weighting factors differently set depending on the neural solution. For static optimization, α and β were set to 1 and 2, respectively, with γ set to 0 (since no experimental EMG data were provided). In contrast, for the EMG-assisted approach the weighting factors were optimized to balance between muscle excitations and joint moments tracking errors (Sartori et al. 2014; Pizzolato et al. 2015), aiming to achieve good EMG data tracking without compromising torque tracking (i.e., large—but not too large—gamma compared to beta values). The weighting factors were therefore set to 1 (α), 1 (β), and 20 (γ). Of note, our static optimization approach differs to familiar implementations such as in OpenSim. In CEINMS (as per Eq. 2), the joint moment-tracking error is minimized as part of the objective function, rather than being incorporated as an optimization constraint. The static optimization approach in CEINMS also enables using an elastic tendon model and the parallel elastic component in the Hill-type muscle model.

Finally, considering the knee joint as planar, at each time point knee JCFs could be estimated solving for the following static equilibrium problem (Winby et al. 2009):

3 $${\text{JCF}}^{{\text{LC/MC}}} = \frac{{\left( {M_{{{\text{MTU}}}}^{{\text{MC/LC}}} - M_{{{\text{ext}}}}^{{\text{MC/LC}}} } \right)}}{{d_{{{\text{IC}}}} }}$$

where ${M}_{\mathrm{ext}}^{\mathrm{MC}/\mathrm{LC}}$ is the external moment around the medial/lateral contact point, ${M}_{\mathrm{MTU}}^{\mathrm{MC}/\mathrm{LC}}$ is the overall muscle torque acting on the medial/lateral knee compartment, and ${d}_{\mathrm{IC}}$ is the intercondylar distance (i.e., between contact points).

## Data analysis

The ability of each model to reproduce physiologically plausible internal biomechanics was assessed by quantifying their ability to track experimental joint moments and EMG data, to predict joint loading within experimentally measured bounds (from instrumented implant data), and to feature or not (MRI-based) personalized MSK anatomies. In particular, the* r*-squared values (*R*^2^) and root-mean-square errors (RMSEs) were computed (1) to compare ankle and knee joint moment estimates from CEINMS to the corresponding OpenSim Inverse Dynamics results and (2) to compare CEINMS predictions and muscle excitations extracted from the experimental surface EMG data. The latter were calculated individually, for each of the 12 lower limb muscles of interest, and reported as mean (standard deviation). The total knee JCFs profiles were quantitatively analyzed to identify any non-physiological loading condition (i.e., exaggerated JCF predictions). A plausibility criterion was selected upon analysis of the recordings of instrumented knee implants (23 trials, 11 patients) collected as part of the Grand Challenge Competition for In vivo Knee Loads (Fregly et al. 2012) and the Orthoload project (Bergmann et al. 2014; Imani Nejad et al. 2020). Briefly, instrumented implant measurements were normalized to BW and interpolated to 101 data points to represent the full gait cycle. In this dataset (*n* = 23 trials), the minimum and maximum BW values for knee JCFs resulted 0 BW and 3.45 BW, respectively. We decided to approximate these values to 0 BW and 3.5 BW, which were selected as lower and upper boundaries for our plausibility criterion. Hence, model-estimated knee JCFs were considered non-physiologically plausible if exceeding 3.5 BW at any time. A scoring system was defined to rank models based on their ability to produce physiologically plausible predictions. One point per model and participant was assigned whenever one of the following criteria was met: (i) good joint moment tracking (*R*^2^ ≥ 0.7), (ii) good muscle excitations tracking ( *R*^2^ ≥ 0.5), (iii) maximal JCF not exceeding 3.5 BW, and (iv) inclusion of accurate musculoskeletal anatomies (from medical images). The model that obtained the highest overall score was considered to be the most physiologically plausible and was thereon used as reference to compare muscle forces, total, medial and lateral JCF estimates. The level of agreement with the most physiologically plausible model was measured computing the* R*^2^ and RMSE values. This enabled the quantification of the individual effect of each level of personalization on the endpoint variables.

## Statistical analysis

Data were checked for normality. If data distributions were normal, a mixed ANOVA was performed to compare *R*^2^ and RMSE values between models and within populations (i.e., TD and CP). Post hoc analyses were conducted using paired t-tests implementing Bonferroni corrections to account for multiple comparisons. If data were not normally distributed, the* R*^2^ and RMSE were compared using a Friedman test for repeated measures followed by a Wilcoxon signed-rank test. Finally, statistical parametrical mapping (SPM) was employed to identify when, during the gait cycle, different models produced significantly different estimates of JCFs. For all tests, statistical significance was initially set to *p* = 0.05. All analyses were conducted in Python 3.6, using the Pingouin (Vallat 2018) and spm1d (Pataky 2012) modules.

## Results

### Assessment of physiological plausibility

**Fig. 1:**
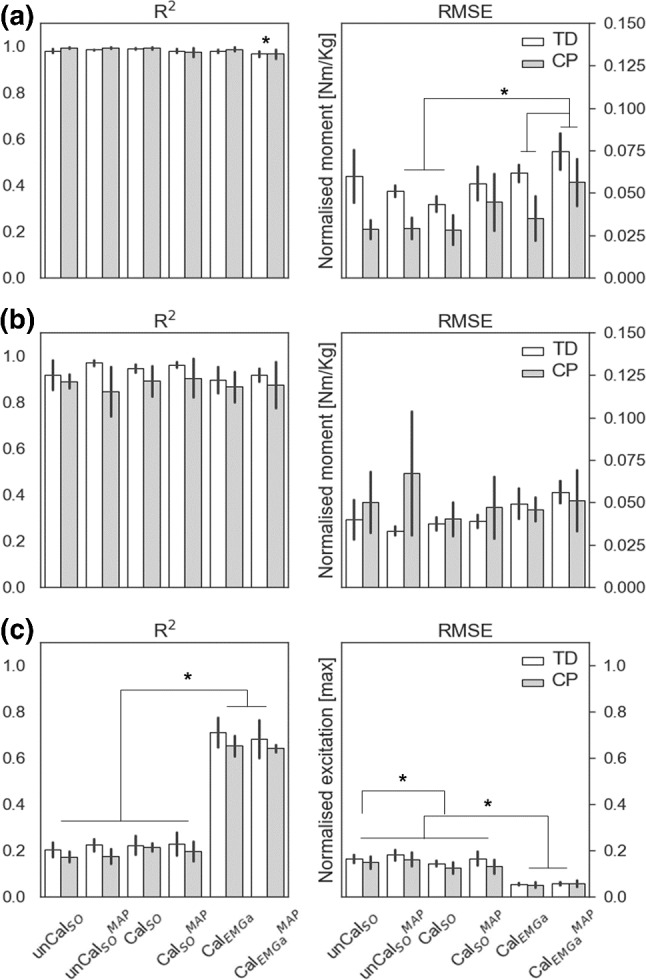
The models’ ability to track experimental data. For each model, *R*^2^ and RMSE values between predicted and experimental ankle joint moment (**a**), knee joint moment (**b**) and EMG data (**c**) were computed. Results are reported as mean across populations, TD (white) and CP (gray). * indicate statistically significant differences (*p* = 0.05)

All models well tracked inverse dynamics’ ankle (*R*^2^ > 0.97 ± 0.02) and knee (*R*^2^ > 0.85 ± 0.13) joint moments (Fig. 1). Significant differences in *R*^2^ values were revealed by the ANOVA test (1) between models (*p* = 0.0015) in tracking the ankle plantarflexion moment and (2) between populations (*p* = 0.047) in tracking the knee flexion moment. Specifically, Cal_EMGa_^MAP^ less accurately tracked ankle joint moments compared to unCal_SO_^MAP^ and Cal_SO_ models, while all six NMSK models better tracked knee joint moments for TD compared to CP participants (on average *R*^2^_TD_ = 0.93 ± 0.05, *R*^2^_CP_ = 0.88 ± 0.08). Low RMSEs (< 0.08 Nm/Kg) were measured for both ankle and knee joint moments, across models and subjects. Ankle plantarflexion tracking errors were significantly lower in pediatric CP models (*p* < 0.0001). Discrepancies between experimental and tracked joint moments were mostly observed around heel strike (first frames) and toe-off (~ 60% gait cycle).

For both TD and CP children, the first four NMSK models, which employed static optimization methods to synthesize muscle excitations, were unable to accurately track experimental EMG data (*R*^2^ < 0.30 ± 0.23, RMSE > 0.09 ± 0.03). Contrarily, Cal_EMGa_ and Cal_EMGa_^MAP^ produced significantly more accurate estimates (*R*^2^ > 0.58 ± 0.25, RMSE < 0.07 ± 0.04, *p* < 0.0001. Fig. 1c).

**Fig. 2:**
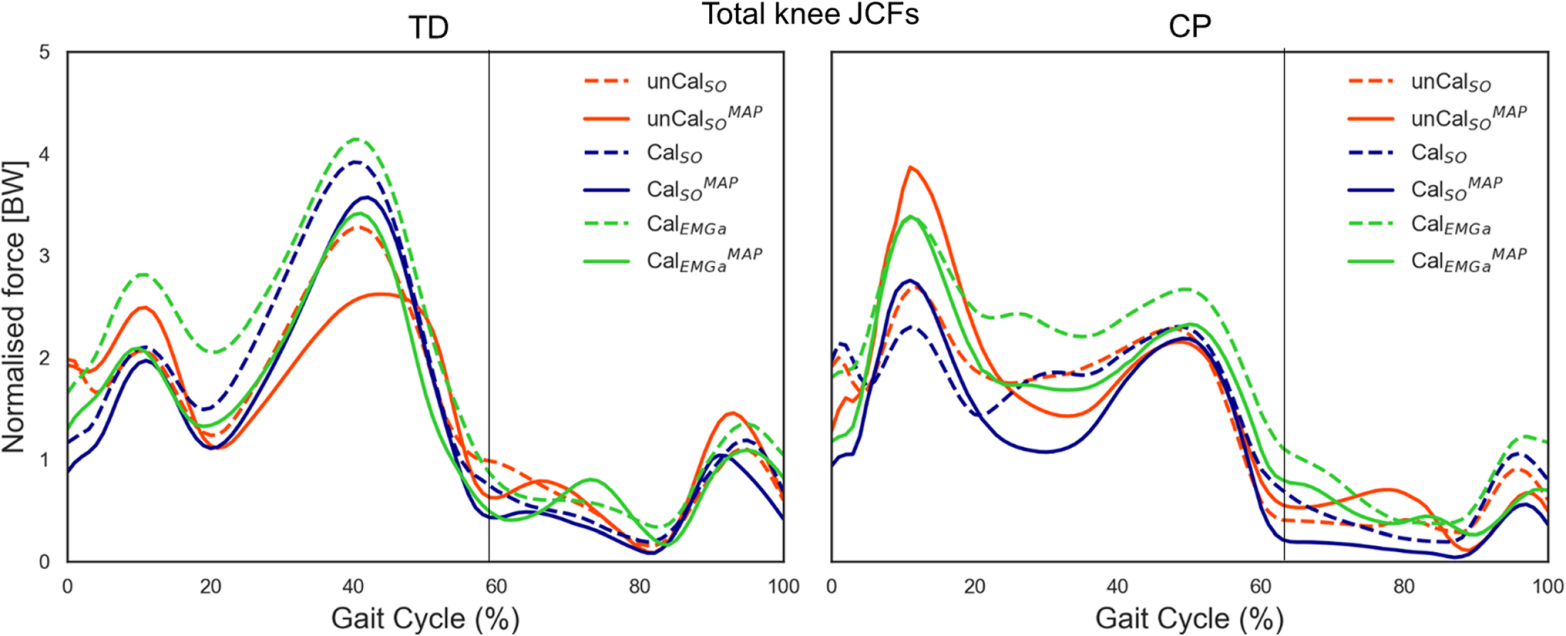
Examples of total knee JCF profiles estimated using the six developed NMSK models, for a TD child and a child with CP. Colors discriminate models based on the definition of musculotendon parameters and neural solution (red = unCal_SO_, blue = Cal_SO_, green = Cal_EMGa_). Line types refer to the implemented musculoskeletal anatomy (solid = personalized in the MAP Client, dashed = scaled generic from OpenSim). The vertical line separates the stance and swing phases of the gait cycle

From a qualitative standpoint, the models with personalized MSK anatomies (superscript MAP) produced lower knee JCF estimates compared to the corresponding scaled generic models, for all children (Fig. 2). Overall, Cal_EMGa_^MAP^ resulted the NMSK model with the highest level of physiological plausibility (Table 4). Therefore, its estimates of muscle forces and knee JCFs were used as reference in subsequent analyses to identify the individual contribution of each personalized feature on models’ outcomes.

**Table 4:** Physiological plausibility score

Model name	Population	Personalized features					
		Tracking moments	Tracking excitations	JCF*	Anatomy	Score	Overall
unCal_SO_	TD CP	•••	-	-	-	3 (4)	7 (9)
		•••	-	•	-	4 (5)	
Cal_SO_	TD CP	•••	-	-	-	3 (4)	7 (9)
		•••	-	•	-	4 (5)	
Cal_EMGa_	TD CP	•••	•••	-	-	6 (6)	13 (14)
		•••	•••	•	-	7 (8)	
unCal_SO_^MAP^	TD CP	•••	-	•	•••	7 (8)	14 (16)
		•••	-	•	•••	7 (8)	
Cal_SO_^MAP^	TD CP	•••	-	••	•••	8 (8)	14 (16)
		•••	-	-	•••	6 (8)	
Cal_EMGa_^MAP^	TD CP	•••	•••	••	•••	11 (11)	**20** (22)
		•••	•••	-	•••	9 (11)	

For each model, a score up to 6 was assigned based on the number of participants (dots) for whom the selected feature/condition was present/met. Population specific, i.e., for typically developing and overall scores per model were calculated. The model associated with the highest score (Cal_EMGa_^MAP^) was considered to be the most physiologically plausible model. *JCF* Joint contact forces

*A JCF plausibility point was assigned to a model only if the condition (0 ≤ total JCF ≤ 3.5 BW) was met for all 10 trials. In brackets is reported the score considering an upper threshold equal to 4 BW (instead of 3.5 BW) for the JCF plausibility

### Individual effect of personalization levels

**Fig. 3:**
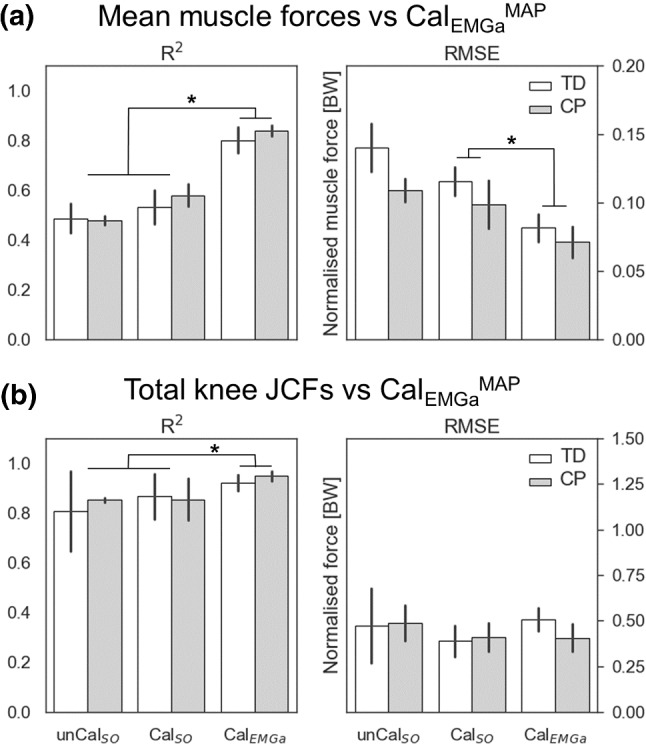
Comparison of **a** muscle forces and **b** total knee JCF estimates between models. The level of personalization was incrementally increased by (1) calibrating musculotendon parameters in CEINMS (unCal_SO_ to Cal_SO_) and (2) switching neural solution (Cal_SO_ to Cal_EMGa_). Estimates from the most physiologically plausible model, i.e., Cal_EMGa_^MAP^, were used as reference, since no experimental data were available. * indicate statistically significant differences (*p* = 0.05)

Increasing the level of personalization was associated with muscle force and JCF profiles more closely approximating the estimates from Cal_EMGa_^MAP^ (Fig. 3). For the muscle forces, calibration of musculotendon parameters only minimally affected the models’ predictions (~ 5%, *p* = 0.104).

However, a ~ 20% significant increment (*p* < 0.001) in *R*^2^ values was observed when switching neural solution from static optimization to an EMG-assisted approach. Similar effects were visible on the RMSE values, which became considerably smaller by using EMG-assisted approaches as opposed to static optimization (*p* < 0.006). Significantly lower RMSEs were obtained in the CP population (*p* = 0.022). The level of personalization also affected total JCF estimates. Switching neural solution to an EMG-assisted approach significantly improved the level of agreement with Cal_EMGa_^MAP^ predictions (*p* = 0.036). On the other hand, calibrating MTU parameters did not lead to significant improvements in the *R*^2^ (*p* = 0.529).

**Fig. 4:**
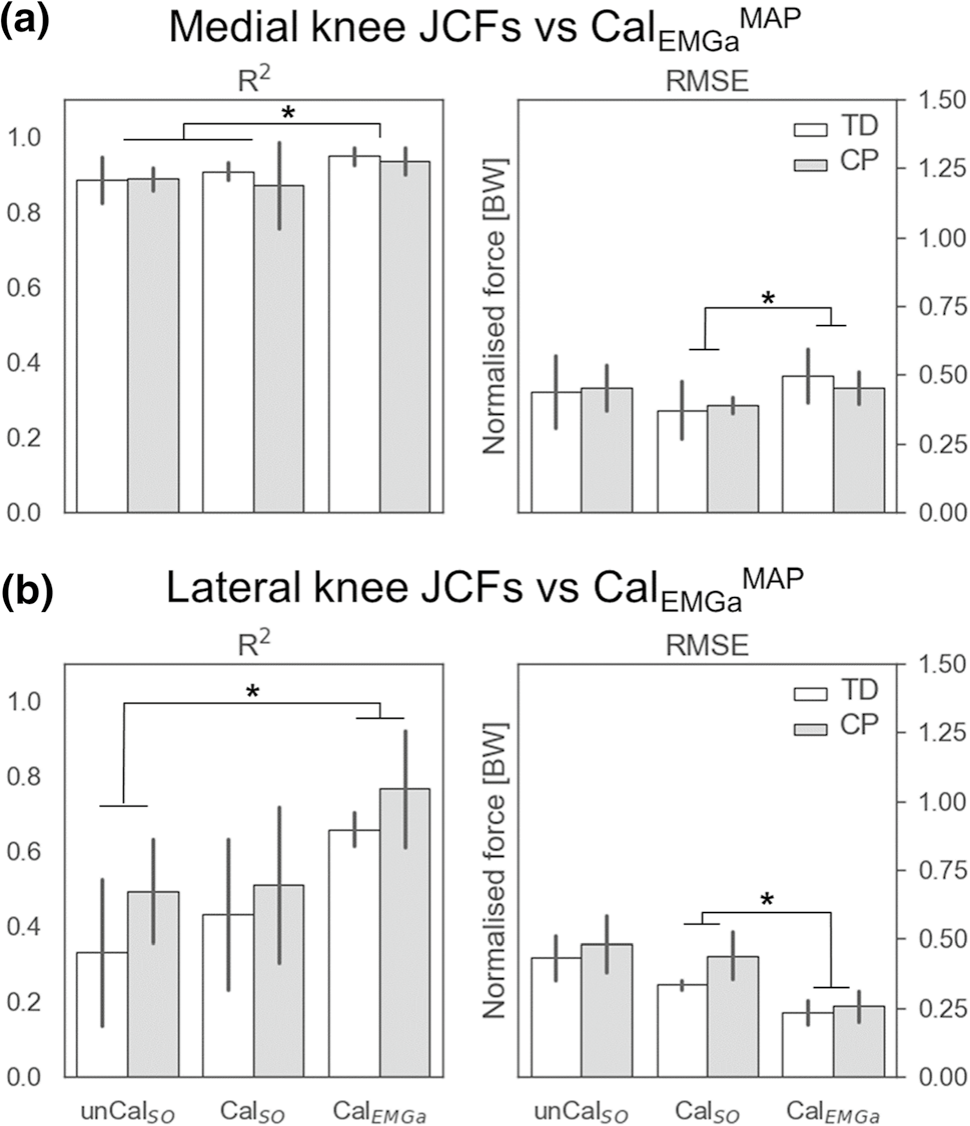
Comparison of **a** medial and **b** lateral JCF estimates between models. The level of personalization was incrementally increased by (1) calibrating musculotendon parameters in CEINMS (unCal_SO_ to Cal_SO_) and (2) switching neural solution (Cal_SO_ to Cal_EMGa_). Estimates from the most physiologically plausible model, i.e., Cal_EMGa_^MAP^, were used as reference, since no experimental data were available. * indicate statistically significant differences (*p* = 0.05)

The agreement between medial JCFs estimated by Cal_EMGa_^MAP^ and the other models was very high (Fig. 4a). The mean *R*^2^ values were larger than 0.87 for all models and across populations, while the RMSE was consistently smaller than 0.5 BW. Nonetheless, unCal_SO_ and Cal_SO_ showed a significantly lower level of agreement with Cal_EMGa_^MAP^ compared to Cal_EMGa_ (*p* = 0.036). However, the RMSEs were smaller for Cal_SO_ than Cal_EMGa_ (*p* < 0.01). On the lateral compartment of the knee joint, the agreement with Cal_EMGa_^MAP^ estimates was lower than on the medial side, for all models (Fig. 4b). The level of personalization had a significant main effect (*p* = 0.006), in which the *R*^2^ was significantly higher using a calibrated EMG-informed model compared to unCal_SO_ (*p* < 0.01) and the RMSE was significantly lower switching from static optimization to EMG-assisted approach (*p* < 0.047). No differences were detected between populations.

**Fig. 5:**
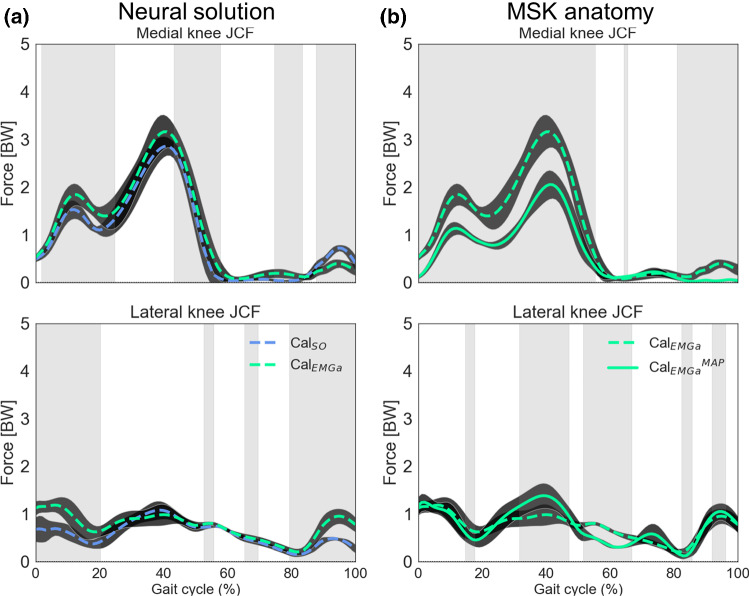
Example of medial and lateral joint contact force estimates for one TD child. Effect of changing neural solution (**a**) and personalizing musculoskeletal anatomy (**b**). Comparisons between Cal_SO_/Cal_EMGa_ and Cal_EMGa_/Cal_EMGa_^MAP^ models, respectively. Gray bands indicate significant differences (*p* < 0.05), as per statistical parametrical mapping (SPM)

Pairwise t-tests in SPM (Fig. 5), conducted separately for each participant, confirmed the above findings. During the stance phase, the lateral compartment was mostly affected by the choice of the neural solution (Fig. 5a), while the medial compartment resulted to be more affected following personalization of musculoskeletal anatomy (Fig. 5b).

## Discussion

For the first time, pediatric NMSK models featuring personalized bony geometries, muscle pathways, MTU parameters and muscle activation patterns were developed and employed to estimate knee JCFs during gait in TD and CP populations. Six different models with incremental level of personalization were developed and compared. Among all, the calibrated EMG-assisted NMSK model with personalized musculoskeletal anatomy produced the most physiologically plausible results. This model accurately tracked experimental joint moments and EMG data and predicted maximal total knee joint contact forces close to instrumented implant data. Assessment of relative model personalization contributions indicated that personalizing anatomy and muscle activation patterns had the largest impact on muscle forces and knee JCF estimates, i.e., our endpoint variables, compared to calibrating MTU parameters.

In agreement with our first hypothesis, the model with the highest level of personalization (Cal_EMGa_^MAP^) produced the most physiologically plausible internal biomechanics (Table 4). Similar to the other developed models, Cal_EMGa_^MAP^ accurately tracked ankle plantar/dorsiflexion, knee flexion/extension, hip flexion/extension and ab/adduction moments (Figs. S1–S6 in Online Resources). In addition, Cal_EMGa_^MAP^ better tracked experimental EMG data compared to models employing static optimization. This is not surprising per se, as the muscle–tendon unit parameters of all Cal_EMGa_^MAP^ models were calibrated using collected EMG data, and the cost function included an EMG-tracking term (in addition to the torque-tracking term, common to all models). Nonetheless, it enables to build trust in the predictions of EMG-assisted models. Indeed, the presence of the EMG-tracking term did not compromise the ability of Cal_EMGa_^MAP^ models to track experimental joint torques. In general, replacing static optimization with an EMG-assisted approach to solve for muscle forces had a significantly larger effect on the ability of models to track EMG data, compared to personalizing the musculoskeletal anatomy (Figs. S7–S8 in Online Resources). Furthermore, Cal_EMGa_^MAP^ predicted total knee joint contact loading more in line with experimental data from instrumented implants (Fregly et al. 2012; Imani Nejad et al. 2020). More specifically, compared to the equivalent generic model employing an EMG-assisted approach (Cal_EMGa_), our most personalized model (Cal_EMGa_^MAP^) produced significantly lower estimates of total knee JCF. This is very important, as previous work (Gerus et al. 2013) showed that generic scaled NMSK models may overestimate in vivo knee JCFs measured with instrumented implants. Finally, Cal_EMGa_^MAP^ was anatomically and physiologically more accurate than Cal_EMGa_ as it featured bony geometries reconstructed from medical imaging data and muscle attachments morphed from the SOMSO anatomical model (compared to generic musculoskeletal anatomies).

Increasing the level of personalization significantly affected muscle force and JCFs estimates. The agreement between Cal_EMGa_^MAP^ and models with a lower level of personalization increased when MTU parameters and neural solution were progressively personalized (Fig. 3). However, some distinctions need to be drawn. Calibrating MTU parameters appeared to have little impact on mean muscle forces, total and medial knee JCF estimates (Figs. 3 and 4). Nonetheless, lateral contact forces were significantly affected (Figs. 2 and 4b). Importantly, switching from static optimization to an EMG-assisted approach considerably improved muscle force tracking (*R*^2^ ~ 0.8, + 20% from Cal_SO_ models. Fig. 3a) and lateral knee JCF tracking (Fig. 4b), and enabled the identification of abnormal profiles in the CP participants. Personalizing the musculoskeletal anatomy had the greatest impact on knee JCFs (Fig. 5b). All models featuring personalized MSK anatomies produced significantly lower JCF estimates than the corresponding generic scaled models (Fig. 5b).

Personalizing the musculoskeletal anatomy resulted in lower JCF estimates, particularly in the medial compartment of the knee joint. This is consistent with previous work, where personalized geometries have been associated with lower estimates of contact force, which more closely approximated experimental data from an instrumented implant (Gerus et al. 2013). In the present study, personalized musculoskeletal anatomies featured image-based bony geometries, muscle attachments morphed from the SOMSO anatomical model, physiologically and physically plausible pathways and MTU kinematics, and personalized joint contact points. Particularly, the location of medial and lateral contact points determined the intercondylar distance, which is denominator in the equation solved to compute JCFs (Eq. 3). For the models with personalized anatomies, the contact points were located where, with the model in default position (i.e., all joint angles set to 0), the Euclidean distance between each tibial plateau and the corresponding femoral condyle was shortest. In contrast, the location of medial and lateral contact points on the scaled generic OpenSim models was defined via regression equations based on adult MRI data (Fig. S9 in Online Resources), resulting in narrower intercondylar distances compared to the MRI-based models (Table S2 in Online Resources) and possibly explaining our research findings on JCF estimates. However, adult and pediatric bones may differ in shape and size. Therefore, what may be valid for an adult population may not be applicable to children (Davico et al. 2020a).

This study had some limitations. First, personalized and generic musculoskeletal anatomies featured two different knee joint mechanisms. However, to reduce kinematics discrepancies between models, which could affect JCF predictions, knee adduction/abduction and internal/external rotation DOFs on the base generic model were locked, to only allow knee flexion/extension. This resulted in comparable knee joint kinematics and kinetics between the developed models (Fig. S10 in Online Resources). Second, three of the six NMSK models developed in this study (i.e., unCal_SO_, Cal_SO_ and Cal_EMGa_) were generated off the gait2392 model, although newer generic musculoskeletal anatomies had become available (e.g., full body model). However, this choice was deemed necessary to enable fair comparisons with the models generated via the MAP Client, which uses the gait2392 as template. Third, the scoring system devised to rank all developed models according to their physiological plausibility was set up to provide an objective assessment of the goodness of a model (and of the modelling pipeline proposed to generate them) but may have not been exhaustive. The selected upper boundary of 3.5 BW used in this study as a criterion for physiologically plausibility was based on experimental measurements on elderly participants with a total knee prosthesis, whose muscle activity may significantly differ from that of a child. This value is, however, in line with a previous computational study that reported predicted JCF values smaller than 3.5 BW both for a healthy pediatric population (max value: 3.0 ± 0.5 BW) and for children with mild CP (max value: 3.2 ± 0.4 BW) (Steele et al. 2012a). Although the body of literature supports the idea that the higher the level of personalization is, the more plausible the estimates are, the authors believe that all these aspects (and potentially more) should always be assessed to build trust in a model. Fourth, although the personalization of MTU pathways aimed to produce physiologically plausible MTU kinematics (i.e., smooth and without discontinuities), the resulting moment arms and MTU lengths curves reflected patterns observed in cadaveric studies. These may not necessarily describe the MTU kinematics of a child. Larger than normal moment arms or increased muscle lengths due to the definition of suboptimal wrapping surfaces may have been produced, which could have affected the final estimates. Nonetheless, visual checks were performed, and inaccuracies were manually corrected. Hence, the use of wrapping surfaces resulted in more physically plausible MTU pathways. In-bone penetrations, which are observed in scaled generic models employing via-points to constrain muscle paths, were removed. However, personalized MTU pathways were not validated against in vivo data. A thorough investigation of the effects of MSK anatomy personalization on MTU kinematics, and consequently on muscle forces and knee JCFs, is yet to be conducted. Fifth, calibration parameters were not standardized for all participants. For TD03 and CP01, who were the smallest children in the studied population (and identical twins), different calibration ranges in CEINMS were used to ensure proper muscle functioning (i.e., to avoid exaggerated muscle force values and abnormal profiles, resulting from inappropriately sized OFL and TSL). Instead of a ± 5% variation, calibration allowed for a ± 10% change for both OFL and TSL values. Sixth, the studied population included only three children per group (CP and TD) due to the amount of work required to develop the 36 NMSK models (Table S3 in Online Resources). Nonetheless, to confirm the research findings and to determine whether increasing the level of model personalization may be more beneficial for pathological populations compared to TD children, the methods proposed in this study should be applied to a larger cohort. Last, although this study primarily focused on the knee joint, we acknowledge that individuals with CP experience altered joint loading at the ankle and hip joints (Morrell et al. 2002). Therefore, a comprehensive evaluation (at the three joints) using the levels of personalization defined in this work would provide a more detailed and informative picture of all CP-related effects on gait. Future work should therefore aim to extend the methods hereby presented to both the ankle and hip joints.

## Conclusions

Personalization is crucial to generate physiologically plausible NMSK models. Particularly, the use of EMG-assisted approaches is recommended to capture atypical muscle activation patterns, while the inclusion of MSK anatomies based on medical imaging data is required to allow for the definition of more anatomically accurate joint geometries and MTU pathways. Moreover, the calibration of MTU parameters should be considered to ensure proper muscle function when working on pediatric populations, especially in the study of neuromuscular disorders. Last, the benefits of personalization become more apparent when multiple features are personalized at once. Future efforts should focus to automate and simplify the generation and use of physiologically plausible NMSK models in clinical practice.

## Supplementary Information

Below is the link to the electronic supplementary material.

Supplementary file1 (DOCX 1610 kb)

## Data Availability

The MAP-Client is freely available (https://map-client.readthedocs.io/en/latest/). More information can be found on the SimTK platform (https://simtk.org/projects/map). The models generated as part of this research are available upon reasonable request.

## References

[CR1] Anderson FC, Pandy MG (2001). Dynamic optimization of human walking. J Biomech Eng.

[CR2] Arnold * AS, Delp SL, (2005) Computer modeling of gait abnormalities in cerebral palsy: application to treatment planning. Theor Issues Ergon Sci 6:305–312. 10.1080/14639220412331329636

[CR3] Bahl JS, Zhang J, Killen BA (2019). Statistical shape modelling versus linear scaling: effects on predictions of hip joint centre location and muscle moment arms in people with hip osteoarthritis. J Biomech.

[CR4] Bergmann G, Bender A, Graichen F (2014). Standardized Loads Acting in Knee Implants. PLoS ONE.

[CR5] Bosmans L, Wesseling M, Desloovere K (2014). Hip contact force in presence of aberrant bone geometry during normal and pathological gait. J Orthop Res.

[CR6] Brito da Luz S, Modenese L, Sancisi N (2017). Feasibility of using MRIs to create subject-specific parallel-mechanism joint models. J Biomech.

[CR7] Brunner R, Romkes J (2008). Abnormal EMG muscle activity during gait in patients without neurological disorders. Gait Posture.

[CR8] Buchanan TS, Shreeve DA (1996). An evaluation of optimization techniques for the prediction of muscle activation patterns during isometric tasks. J Biomech Eng.

[CR9] Carbone V (2016) Subject-specific lower extremity modeling: personalization of musculoskeletal models using medical imaging and functional measurements. PhD dissertation, University of Twente

[CR10] Correa TA, Pandy MG (2011). A mass-length scaling law for modeling muscle strength in the lower limb. J Biomech.

[CR11] Crowninshield RD, Brand RA (1981). A physiologically based criterion of muscle force prediction in locomotion. J Biomech.

[CR12] Davico G, Pizzolato C, Killen BA (2020). Best methods and data to reconstruct paediatric lower limb bones for musculoskeletal modelling. Biomech Model Mechanobiol.

[CR13] Davico G, Pizzolato C, Lloyd DG (2020). Increasing level of neuromusculoskeletal model personalisation to investigate joint contact forces in cerebral palsy: a twin case study. Clin Biomech.

[CR14] Delp SL, Loan JP, Hoy MG (1990). An interactive graphics-based model of the lower extremity to study orthopaedic surgical procedures. IEEE Trans Biomed Eng.

[CR15] Delp SL, Anderson FC, Arnold AS (2007). OpenSim: open-source software to create and analyze dynamic simulations of movement. IEEE Trans Biomed Eng.

[CR16] Devaprakash D, Weir GJ, Dunne JJ (2016). The influence of digital filter type, amplitude normalisation method, and co-contraction algorithm on clinically relevant surface electromyography data during clinical movement assessments. J Electromyogr Kinesiol.

[CR17] Fox MD, Reinbolt JA, Õunpuu S, Delp SL (2009). Mechanisms of improved knee flexion after rectus femoris transfer surgery. J Biomech.

[CR18] Fox AS, Carty CP, Modenese L (2018). Simulating the effect of muscle weakness and contracture on neuromuscular control of normal gait in children. Gait Posture.

[CR19] Fregly BJ, Besier TF, Lloyd DG (2012). Grand challenge competition to predict in vivo knee loads. J Orthop Res.

[CR20] Gerus P, Sartori M, Besier TF (2013). Subject-specific knee joint geometry improves predictions of medial tibiofemoral contact forces. J Biomech.

[CR21] Herzog W, Read LJ, ter Keurs HEDJ (1991). Experimental determination of force—length relations of intact human gastrocnemius muscles. Clin Biomech.

[CR22] Hoang HX, Pizzolato C, Diamond LE, Lloyd DG (2018). Subject-specific calibration of neuromuscular parameters enables neuromusculoskeletal models to estimate physiologically plausible hip joint contact forces in healthy adults. J Biomech.

[CR23] Imani Nejad Z, Khalili K, Hosseini Nasab SH (2020). The capacity of generic musculoskeletal simulations to predict knee joint loading using the CAMS-knee datasets. Ann Biomed Eng.

[CR24] Kainz H, Graham D, Edwards J (2017). Reliability of four models for clinical gait analysis. Gait Posture.

[CR25] Kainz H, Hoang HX, Stockton C (2017). Accuracy and reliability of marker-based approaches to scale the pelvis, thigh, and shank segments in musculoskeletal models. J Appl Biomech.

[CR26] Killen BA, Brito da Luz S, Lloyd DG (2020). Automated creation and tuning of personalised muscle paths for OpenSim musculoskeletal models of the knee joint. Biomech Model Mechanobiol.

[CR27] Killen BA, Falisse A, De Groote F, Jonkers I (2020). In silico-enhanced treatment and rehabilitation planning for patients with musculoskeletal disorders: can musculoskeletal modelling and dynamic simulations really impact current clinical practice?. Appl Sci.

[CR28] Lenaerts G, De Groote F, Demeulenaere B (2008). Subject-specific hip geometry affects predicted hip joint contact forces during gait. J Biomech.

[CR29] Lloyd DG, Besier TF (2003). An EMG-driven musculoskeletal model to estimate muscle forces and knee joint moments in vivo. J Biomech.

[CR30] Mantoan A, Pizzolato C, Sartori M (2015). MOtoNMS: A MATLAB toolbox to process motion data for neuromusculoskeletal modeling and simulation. Source Code Biol Med.

[CR31] Meyer AJ, Patten C, Fregly BJ (2017). Lower extremity EMG-driven modeling of walking with automated adjustment of musculoskeletal geometry. PLoS ONE.

[CR32] Modenese L, Ceseracciu E, Reggiani M, Lloyd DG (2016). Estimation of musculotendon parameters for scaled and subject specific musculoskeletal models using an optimization technique. J Biomech.

[CR33] Modenese L, Montefiori E, Wang A (2018). Investigation of the dependence of joint contact forces on musculotendon parameters using a codified workflow for image-based modelling. J Biomech.

[CR34] Morrell DS, Pearson JM, Sauser DD (2002). Progressive bone and joint abnormalities of the spine and lower extremities in cerebral palsy. Radiographics.

[CR35] Noble JJ, Chruscikowski E, Fry NRD (2017). The relationship between lower limb muscle volume and body mass in ambulant individuals with bilateral cerebral palsy. BMC Neurol.

[CR36] Nolte D, Tsang CK, Zhang KY (2016). Non-linear scaling of a musculoskeletal model of the lower limb using statistical shape models. J Biomech.

[CR37] Oberhofer K, Lorenzetti S, Mithraratne K (2019) Host Mesh Fitting of a Generic Musculoskeletal Model of the Lower Limbs to Subject-Specific Body Surface Data: A Validation Study. Appl. Bionics Biomech. 10.1155/2019/8381351 PMC6398081 30906423

[CR38] Pataky TC (2012). One-dimensional statistical parametric mapping in Python. Comput Methods Biomech Biomed Engin.

[CR200] Gerus P,  Rao G,  Berton E (2015). Ultrasound-based subject-specific parameters improve fascicle behaviour estimation in Hill-type muscle model. Comput Methods Biomech Biomed Engin.

[CR39] Pellikaan P, van der Krogt MM, Carbone V (2014). Evaluation of a morphing based method to estimate muscle attachment sites of the lower extremity. J Biomech.

[CR40] Pitto L, Kainz H, Falisse A et al (2019) SimCP: a simulation platform to predict gait performance following orthopedic intervention in children with cerebral palsy. Front Neurorobotics 13. 10.3389/fnbot.2019.00054 10.3389/fnbot.2019.00054 PMC6650580 31379550

[CR41] Pizzolato C, Lloyd DG, Sartori M (2015). CEINMS: a toolbox to investigate the influence of different neural control solutions on the prediction of muscle excitation and joint moments during dynamic motor tasks. J Biomech.

[CR42] Sartori M, Reggiani M, Farina D, Lloyd DG (2012). EMG-driven forward-dynamic estimation of muscle force and joint moment about multiple degrees of freedom in the human lower extremity. PLoS ONE.

[CR43] Sartori M, Farina D, Lloyd DG (2014). Hybrid neuromusculoskeletal modeling to best track joint moments using a balance between muscle excitations derived from electromyograms and optimization. J Biomech.

[CR44] Saxby DJ, Modenese L, Bryant AL (2016). Tibiofemoral contact forces during walking, running and sidestepping. Gait Posture.

[CR45] Saxby DJ, Killen BA, Pizzolato C (2020). Machine learning methods to support personalized neuromusculoskeletal modelling. Biomech Model Mechanobiol.

[CR46] Scheys L, Van Campenhout A, Spaepen A (2008). Personalized MR-based musculoskeletal models compared to rescaled generic models in the presence of increased femoral anteversion: Effect on hip moment arm lengths. Gait Posture.

[CR47] Serrancolí G, Kinney AL, Fregly BJ, Font-Llagunes JM (2016). Neuromusculoskeletal model calibration significantly affects predicted knee contact forces for walking. J Biomech Eng.

[CR48] Steele KM, DeMers MS, Schwartz MH, Delp SL (2012). Compressive tibiofemoral force during crouch gait. Gait Posture.

[CR49] Steele KM, van der Krogt MM, Schwartz MH, Delp SL (2012). How much muscle strength is required to walk in a crouch gait?. J Biomech.

[CR50] Suwarganda EK, Diamond LE, Lloyd DG (2019). Minimal medical imaging can accurately reconstruct geometric bone models for musculoskeletal models. PLoS ONE.

[CR51] Thelen DG, Anderson FC, Delp SL (2003). Generating dynamic simulations of movement using computed muscle control. J Biomech.

[CR52] Vallat R (2018) Pingouin: statistics in Python. J Open Source Softw 3:1026. 10.21105/joss.01026

[CR53] van der Krogt MM, Bar-On L, Kindt T (2016). Neuro-musculoskeletal simulation of instrumented contracture and spasticity assessment in children with cerebral palsy. J NeuroEngineering Rehabil.

[CR54] van Veen BC, Mazzà C, Viceconti M (2020) The uncontrolled manifold theory could explain part of the inter-trial variability of knee contact force during level walking. IEEE Trans Neural Syst Rehabil Eng 1–1. 10.1109/TNSRE.2020.3003559 10.1109/TNSRE.2020.3003559 32746299

[CR55] Veerkamp K, Schallig W, Harlaar J (2019). The effects of electromyography-assisted modelling in estimating musculotendon forces during gait in children with cerebral palsy. J Biomech.

[CR56] Ward SR, Kingsbury T, Winters T (2007). Scaling of joint mechanics and muscle architecture in the human knee.

[CR57] Ward SR, Smallwood LH, Lieber RL (2005) Scaling of human lower extremity muscle architecture to skeletal dimensions. Cleveland, OH, USA

[CR58] Wesseling M, Bosmans L, Dijck CV (2019). Non-rigid deformation to include subject-specific detail in musculoskeletal models of CP children with proximal femoral deformity and its effect on muscle and contact forces during gait. Comput Methods Biomech Biomed Engin.

[CR59] Winby CR, Lloyd DG, Kirk TB (2008). Evaluation of different analytical methods for subject-specific scaling of musculotendon parameters. J Biomech.

[CR60] Winby CR, Lloyd DG, Besier TF, Kirk TB (2009). Muscle and external load contribution to knee joint contact loads during normal gait. J Biomech.

[CR61] Zhang J, Sorby H, Clement J, Bello F, Cotin S (2014). The MAP client: user-friendly musculoskeletal modelling workflows. Biomedical Simulation.

