# Supplementary material for: Multi-level personalization of neuromusculoskeletal models to estimate physiologically plausible knee joint contact forces in children
Source: Biomech Model Mechanobiol. 2022 Oct 13;21(6):1873–86. doi: 10.1007/s10237-022-01626-w (PMC9700656; doi:10.1007/s10237-022-01626-w)
Supplement: Supplementary file 1 — Supplementary file1 (DOCX 1610 kb) [file 10237_2022_1626_MOESM1_ESM.docx]

**Supplementary material**

The Supplementary material presents additional data including details on the wrapping surfaces included in the models generated via the MAP client (Table S1), the ability of all models to accurately track experimental hip, knee and ankle joint moments (Fig. S1-6), the correspondence between experimental EMG data (muscle excitations) and estimated muscle force during level walking for one child with CP and one TD participant (Fig. S7-8), the anatomical differences possibly contributing to the findings on knee joint contact forces (Fig. S9, Table S2), and the level of agreement in joint kinematics and kinetics between models featuring generic and personalized musculoskeletal anatomies (Fig. S10). Last, Table S3 shows the time expenditure to generate each of the developed models.

**Table S1.** List of wrapping surfaces introduced in all MAP client generated models to avoid non-physiological muscle tendon unit behaviour (i.e., in-bone penetration at certain joint angles).
PS = psoas, IL = iliacus, Gmax = Gluteus maximus, VM/I/L = vastus medialisKnee/intermedius/lateralis, TFL = tensor fascia lata, SART = sartorius, RF = rectus femoris, M/LG = medial/lateral gastrocnemius, BFL/SH = Biceps femoris long/short head, ST = semitendinosus, SM = semimembranosus. Locations: ant = anterior, pos = posterior, med = medial, lat = lateral.

| Body | Joint | Muscle(s) | Type |
| --- | --- | --- | --- |
| Pelvis | Hip | PS, IL, Gmax | ellipsoid |
| Femur (shaft) | - | VM, VL, VI, TFL | cylinder |
| Femur (neck-shaft) | - | SART, TFL (x2) | cylinder |
| Femur (head) | - | RF | cylinder |
| Femur (condyles) – ant | Knee | VM, VL, VI, RF | cylinder |
| Femur (condyles) – post | Knee | SART, MG, LG | cylinder |
| Femur (condyles) - lat | Knee | VL, BFLH, BFSH | sphere |
| Femur (condyles) - med | Knee | SM, ST, SART, GRAC | sphere |
| Femur | Knee | SART | cylinder |
| Tibia (condyles) | Knee | VM, VL, VI, RF | cylinder |
| Tibia (condyles) - med | Knee | VM, VL, VI, RF, ST, MG, GRAC, SART | sphere |
| Tibia (condyles) - med | Knee | SM, ST (x2), SART (x2), GRAC (x2) | cylinder |
| Tibia (condyles) - lat | Knee | TFL | cylinder |
| Tibia - post | - | MG (x2), LG (x2), BFLH, BFSH | cylinder |


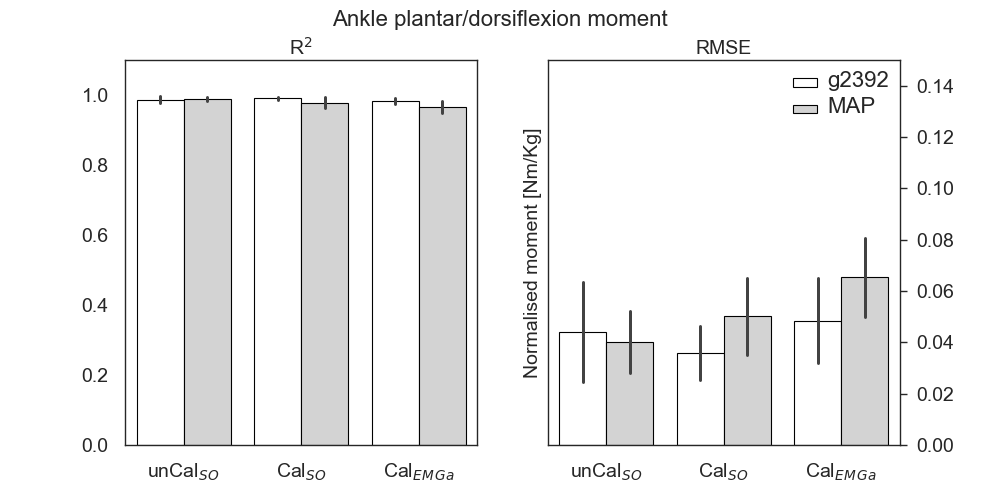
**Fig S1.** Level of agreement between experimental (OpenSim) and predicted (CEINMS) ankle plantar/dorsifelxion moments for the six developed models. The coefficient of determination (R^2^) and root mean square error (RMSE) are reported as mean values across the analyzed cohort (n=6 children). Colors discriminate between musculoskeletal anatomies (white = gait2392 generic model, gray = MAP client generated model). unCal_SO_ = models feat. linearly and morphometrically scaled MTU parameters, employing static optimization; Cal_SO_ = models feat. calibrated MTU parameters, emplpying Static Optimisation, Cal_EMGa_ = models feat. calibrated MTU parameters and employing an EMG-assisted approach.


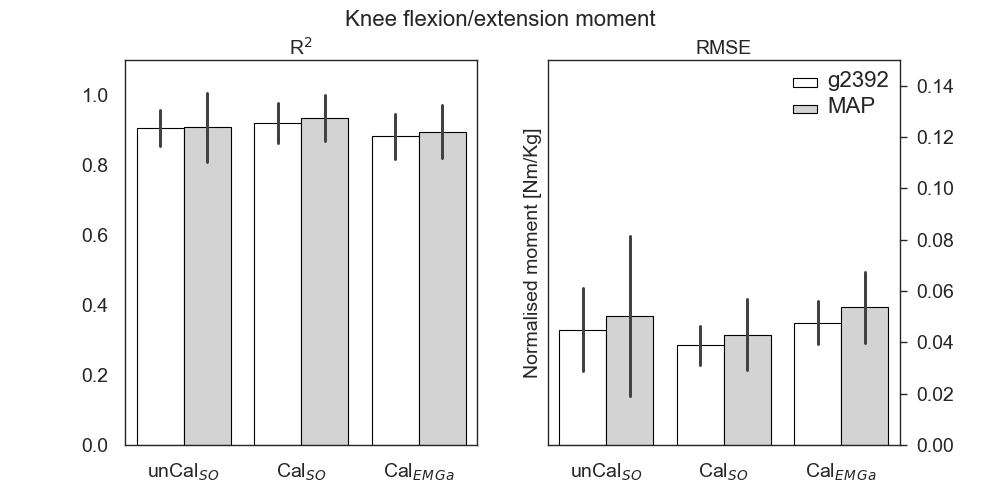
**Fig S2.** Level of agreement between experimental (OpenSim) and predicted (CEINMS) knee flexion/extension moments for the six developed models. The coefficient of determination (R^2^) and root mean square error (RMSE) are reported as mean values across the analyzed cohort (n=6 children). Colors discriminate between musculoskeletal anatomies (white = gait2392 generic model, gray = MAP client generated model). unCal_SO_ = models feat. linearly and morphometrically scaled MTU parameters, employing static optimization; Cal_SO_ = models feat. calibrated MTU parameters, emplpying Static Optimisation, Cal_EMGa_ = models feat. calibrated MTU parameters and employing an EMG-assisted approach.


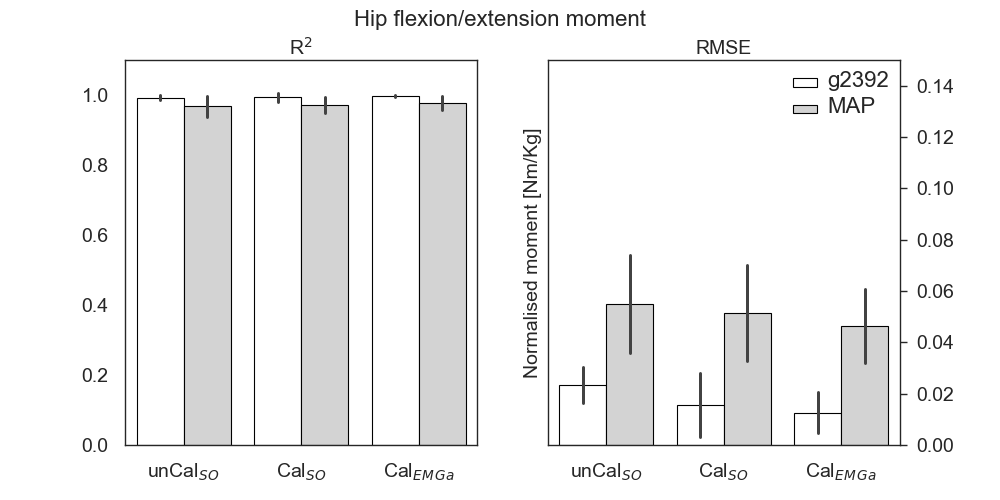
**Fig S3.** Level of agreement between experimental (OpenSim) and predicted (CEINMS) hip flexion/extension moments for the six developed models. The coefficient of determination (R^2^) and root mean square error (RMSE) are reported as mean values across the analyzed cohort (n=6 children). Colors discriminate between musculoskeletal anatomies (white = gait2392 generic model, gray = MAP client generated model). unCal_SO_ = models feat. linearly and morphometrically scaled MTU parameters, employing static optimization; Cal_SO_ = models feat. calibrated MTU parameters, emplpying Static Optimisation, Cal_EMGa_ = models feat. calibrated MTU parameters and employing an EMG-assisted approach.


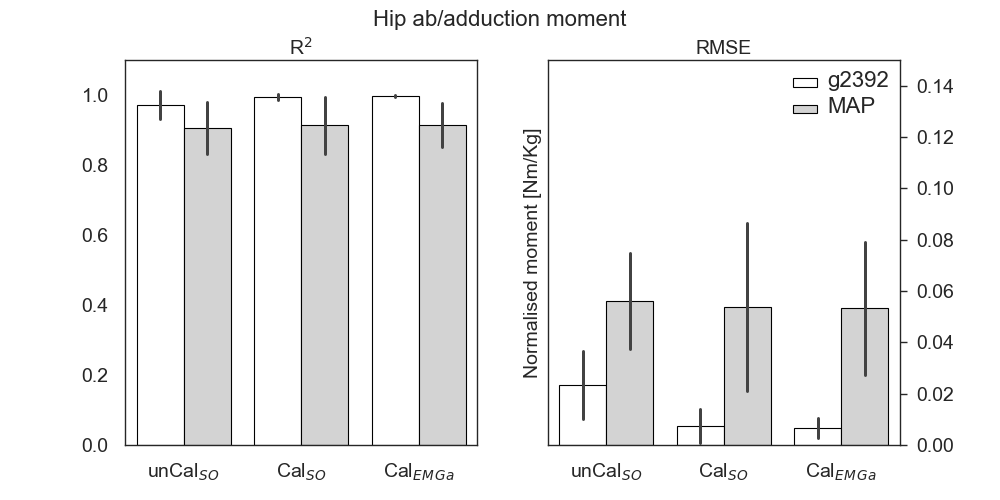
**Fig S4.** Level of agreement between experimental (OpenSim) and predicted (CEINMS) hip internal/external rotation moments for the six developed models. The coefficient of determination (R^2^) and root mean square error (RMSE) are reported as mean values across the analyzed cohort (n=6 children). Colors discriminate between musculoskeletal anatomies (white = gait2392 generic model, gray = MAP client generated model). unCal_SO_ = models feat. linearly and morphometrically scaled MTU parameters, employing static optimization; Cal_SO_ = models feat. calibrated MTU parameters, emplpying Static Optimisation, Cal_EMGa_ = models feat. calibrated MTU parameters and employing an EMG-assisted approach.

**Fig S5.** Comparison between hip, knee and ankle joint moments profiles for one child with CP. On the left side, results from models featuring generic musculoskeletal anatomies (from the gait2392 model). On the right side, results from models featuring personalised musculoskeletal anatomies, reconstructed from magnetic resonance imaging data of the participant under study. Colors discriminate between models. unCal_SO_ = models feat. linearly and morphometrically scaled MTU parameters, employing static optimization; Cal_SO_ = models feat. calibrated MTU parameters, emplpying Static Optimisation, Cal_EMGa_ = models feat. calibrated MTU parameters and employing an EMG-assisted approach.


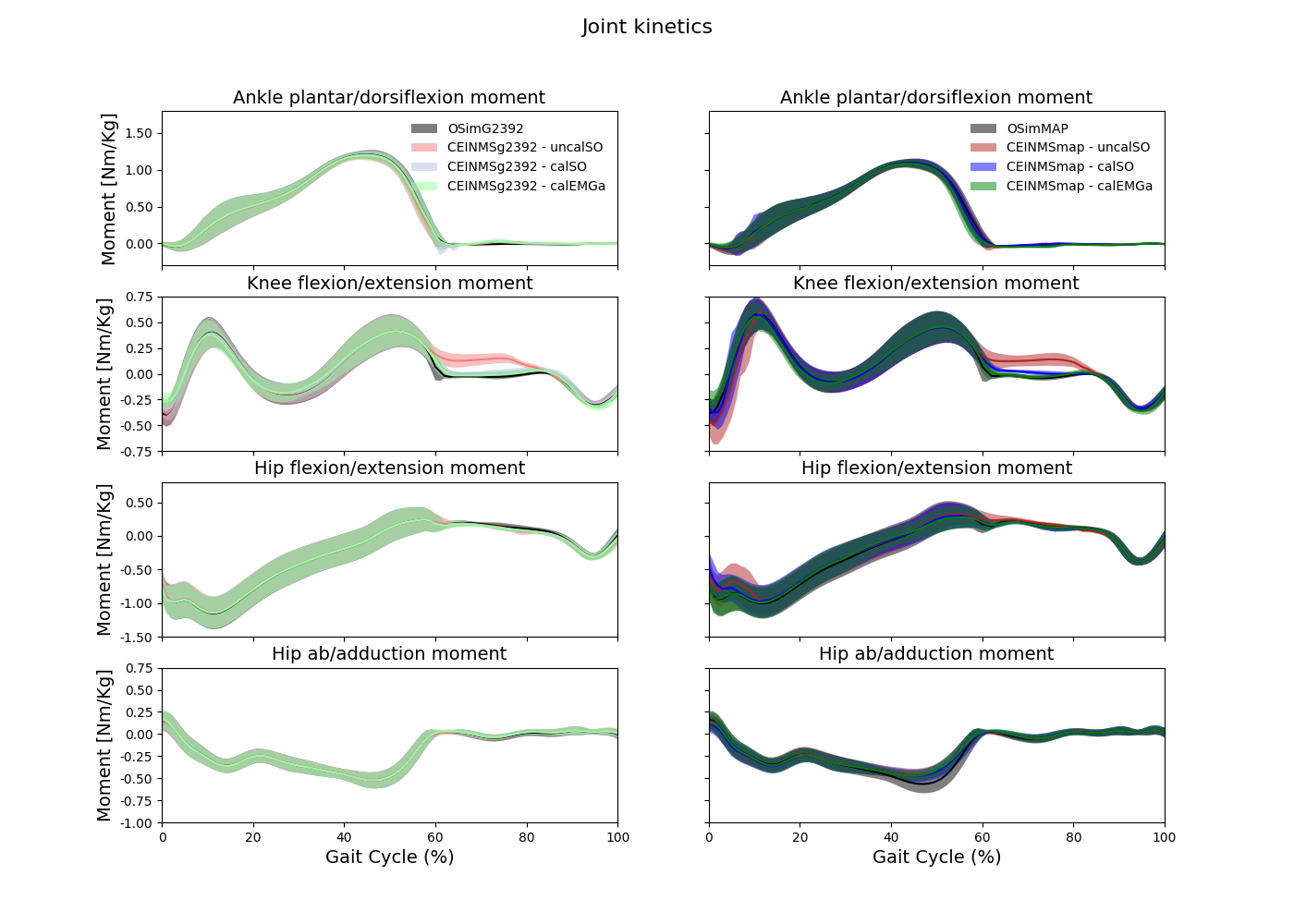


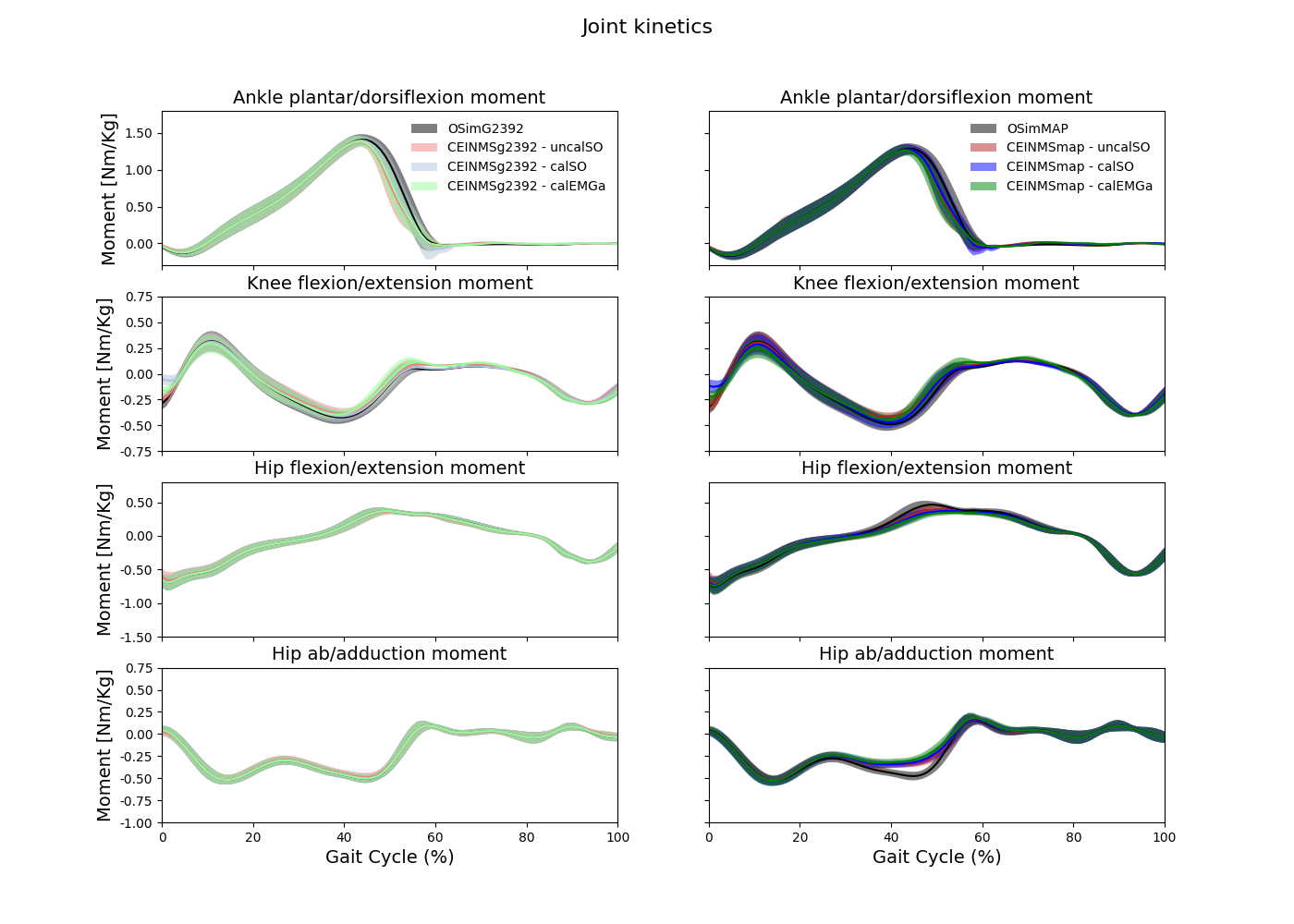
**Fig S6.** Comparison between hip, knee and ankle joint moments profiles for one TD child. On the left side, results from models featuring generic musculoskeletal anatomies (from the gait2392 model). On the right side, results from models featuring personalised musculoskeletal anatomies, reconstructed from magnetic resonance imaging data of the participant under study. Colors discriminate between models. unCal_SO_ = models feat. linearly and morphometrically scaled MTU parameters, employing static optimization; Cal_SO_ = models feat. calibrated MTU parameters, emplpying Static Optimisation, Cal_EMGa_ = models feat. calibrated MTU parameters and employing an EMG-assisted approach.


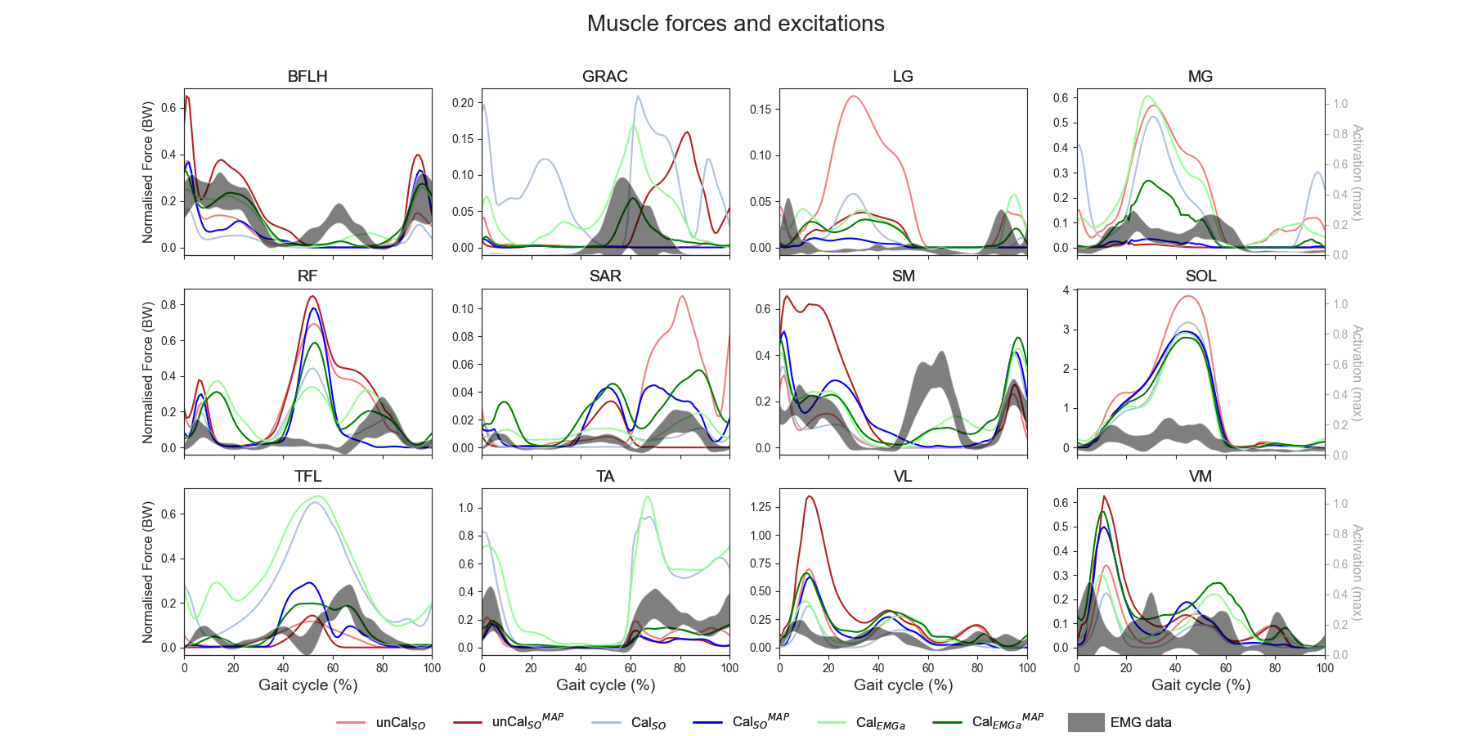
**Fig S7.** Predicted muscle forces, overlayed to the corresponding experimental electromyographic (EMG) data for one child with CP. Models’ estimates are reported as mean across trials (n=10), with colors to discriminate between models. The gray bands represent the mean(±standard deviation) EMG signals recorded during level walking. BFLH = biceps femoris long head, GRAC = gracilis, LG = lateral gastrocnemius, MG = medial gastrocnemius, RF = rectus femoris, SAR = sartorius, SM = Semimembranosus, SOL = soleus, TFL = tensor fascia lata, TA = tibialis anterior, VL = vastus lateralis, VM = vastus medialis.


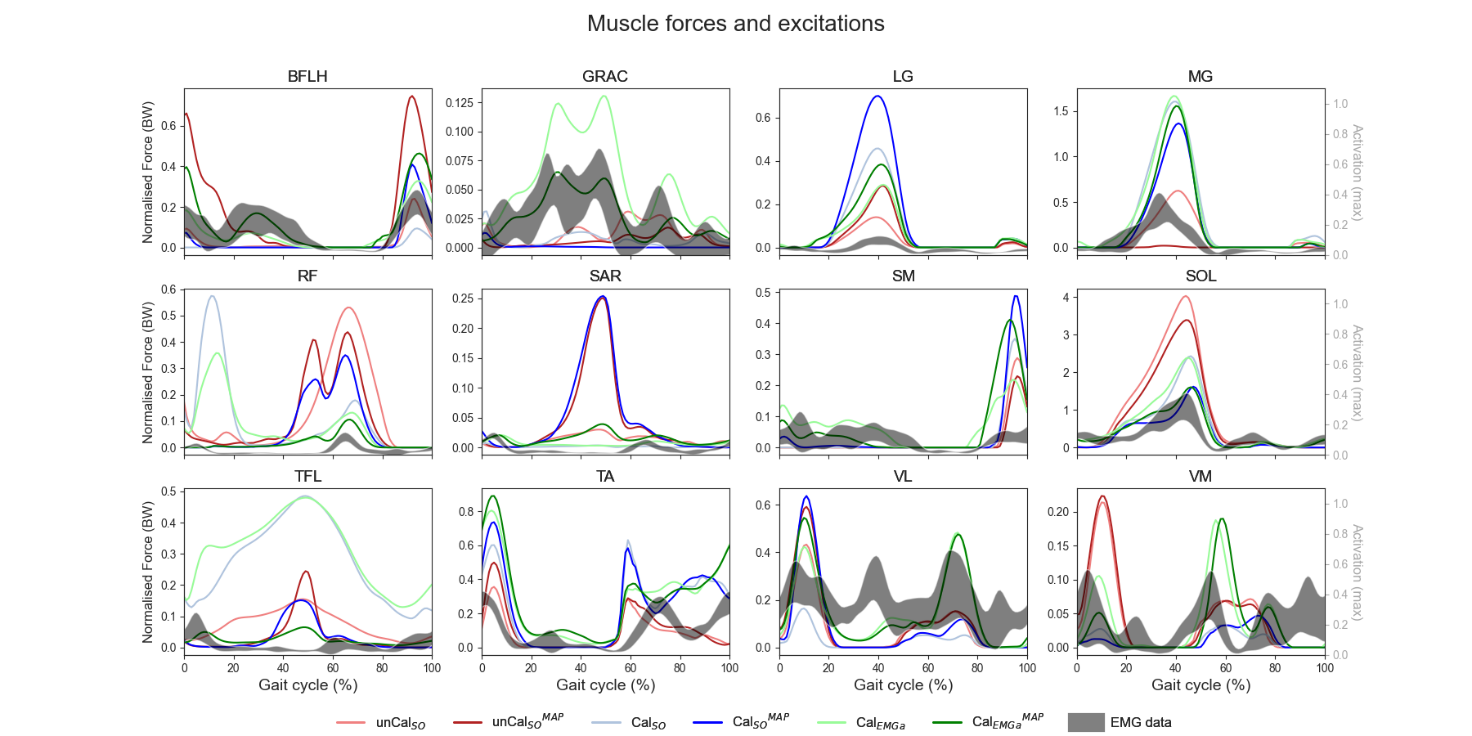
**Fig S8.** Predicted muscle forces, overlayed to the corresponding experimental electromyographic (EMG) data for one TD child. Models’ estimates are reported as mean across trials (n=10), with colors to discriminate between models. The gray bands represent the mean(±standard deviation) EMG signals recorded during level walking. BFLH = biceps femoris long head, GRAC = gracilis, LG = lateral gastrocnemius, MG = medial gastrocnemius, RF = rectus femoris, SAR = sartorius, SM = Semimembranosus, SOL = soleus, TFL = tensor fascia lata, TA = tibialis anterior, VL = vastus lateralis, VM = vastus medialis.

**Fig. S9.** Anatomical differences between models. Personalized (a) versus scaled generic (b) anatomy showing how the intercondylar distance, i.e., distance between medial and lateral contact points, differed depending on the implemented musculoskeletal anatomy.


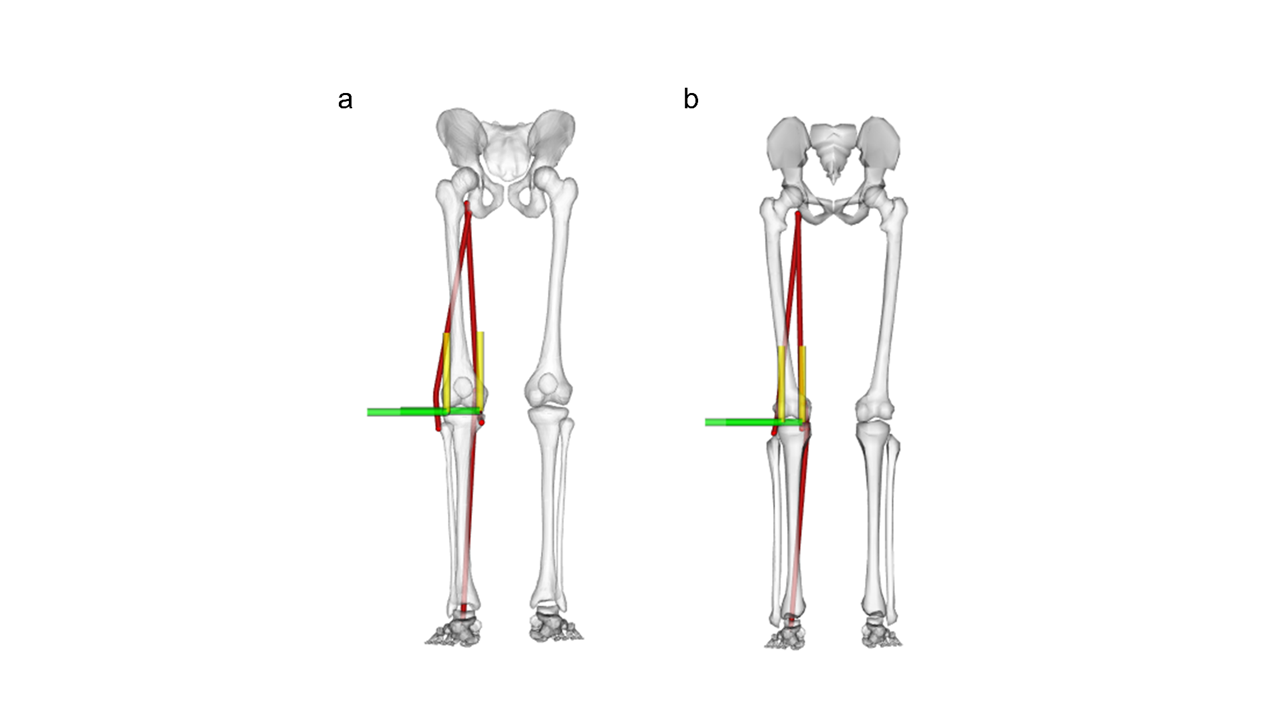


**Table S2.** Intercondylar distances. Intercondylar distance, in generic scaled and personalized anatomies. All values are reported in meters.

| Model anatomy | Subject | | | | | | | | | | | |
| --- | --- | --- | --- | --- | --- | --- | --- | --- | --- | --- | --- | --- |
|  | TD01 | | TD02 | | TD03 | | CP01 | | CP02 | | CP03 | |
| Generic | | 0.033 | 0.027 | | 0.026 | | 0.023 | | 0.036 | | 0.031 | |
| MAP | | 0.053 | 0.045 | | 0.046 | | 0.043 | | 0.057 | | 0.049 | |


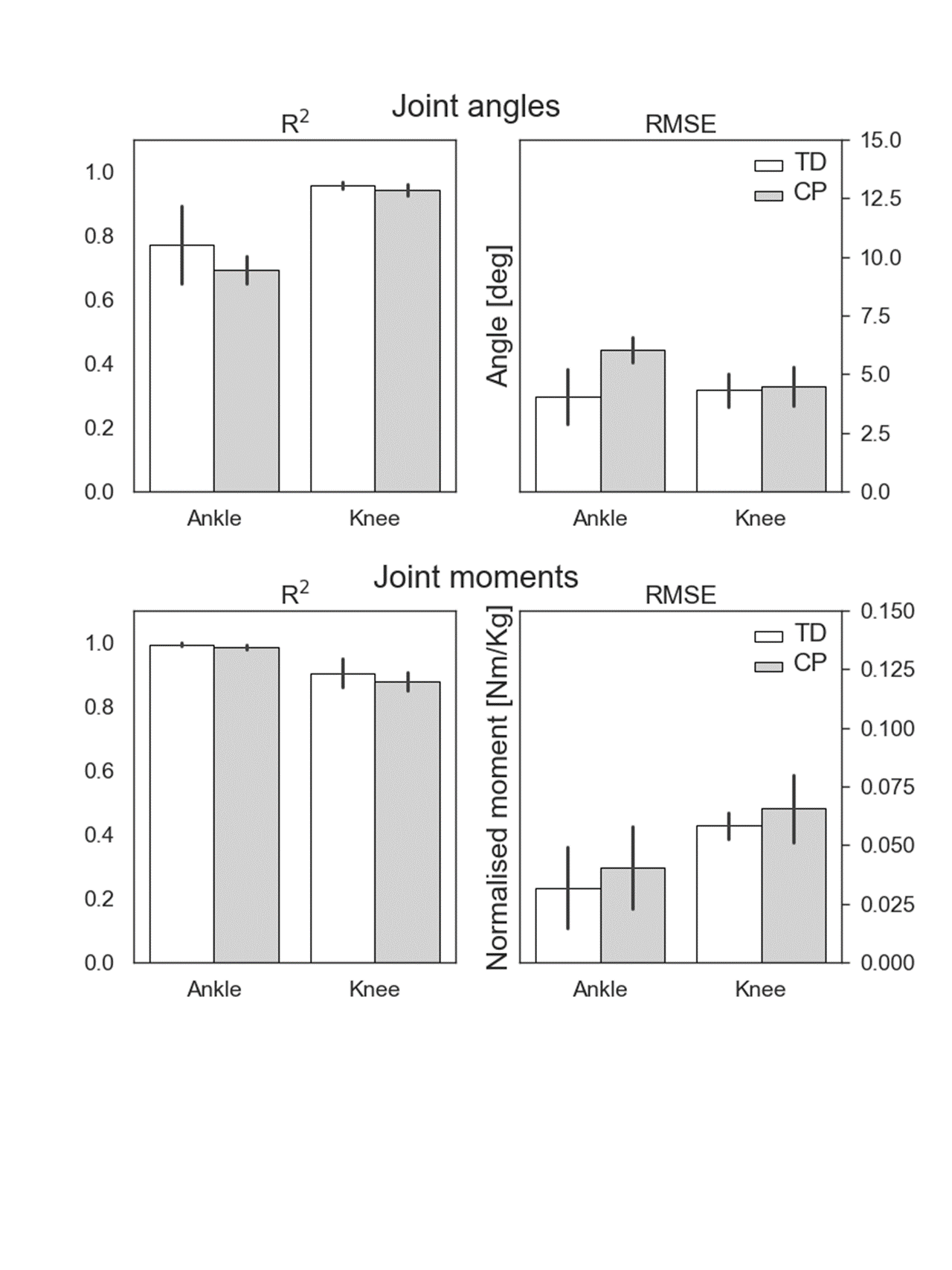
**Fig S10.** Kinematics and kinetics differences between models. Joint angles and moments comparison between a generic scaled OpenSim models and models with personalized musculoskeletal anatomies. The level of agreement was quantified computing the R^2^ and root mean square error (RMSE) between models’ estimates.

|  | **Bone generation** | | **Model creation** | | **MTU parameters**  **tuning and calibration** | | | **Overall** |
| --- | --- | --- | --- | --- | --- | --- | --- | --- |
|  | **Comp.** | **Manual** | **Comp.** | **Manual** | **Comp.** | | **Manual** |  |
| **unCal_SO_** | - | - | 10-15’ | - | 1 h  (MorphSc) | | - | 1h15’ |
| **Cal_SO_** | - | - | 10-15’ | - | 1 h  (MorphSc) | 1 h* (CEINMS) | 15’ | 2h30’ |
| **Cal_EMGa_** | - | - | 10-15’ | - | 1 h  (MorphSc) | 1 h* (CEINMS) | 15’ | 2h30’ |
| **unCal_SO_^MAP^** | 1-5h*  (MAP) | 2 h  (segm.) | 1 h  (Python) | 1-2 h  (checks) | 1 h  (MorphSc) | | - | 7-11h |
| **Cal_SO_^MAP^** |  |  |  |  | 1 h  (MorphSc) | 1 h* (CEINMS) | 15’ | 8-12h |
| **Cal_EMGa_^MAP^** |  |  |  |  | 1 h  (MorphSc) | 1 h* (CEINMS) | 15’ | 8-12h |
| *depending on the selected pipeline to develop bone models | | | | | | | | |

**Table S3.** Time required to develop each of the six musculoskeletal models employed in this study. Computational and manual work are reported separately. unCal_SO_ = models feat. linearly and morphometrically scaled MTU parameters, employing static optimization; cal_SO_ = models feat. calibrated MTU parameters, emplpying Static Optimisation, cal_EMGa_ = models feat. calibrated MTU parameters and employing an EMG-assisted approach. MorphSc = Morphometric scaling in MATLAB; MAP = MAP client; segm. = bone segmentations on MRI data
